# Comparing the Effects of Adequate and Insufficient Sleep on the Mental Health, Behavioral Outcomes, and Daily Lives of South Korean Adolescents

**DOI:** 10.3390/healthcare13050471

**Published:** 2025-02-21

**Authors:** Sang Mi Kim, Hye Seon Park, Yeong Mi Jeong, Catherine Park

**Affiliations:** 1 Department of AI Health Information, Yonsei University, Wonju 26493, Republic of Korea; seasea12@yonsei.ac.kr; 2 Department of Social Welfare and Childcare, Gyeongnam Geochang University, Geochang 50147, Republic of Korea; zzing65@hanmail.net (H.S.P.); mongsil57@hanmail.net (Y.M.J.); 3 Division of Digital Healthcare, Yonsei University, Wonju 26493, Republic of Korea

**Keywords:** adolescent sleep, mental health, health behaviors, academic performance, smartphone addiction, South Korea

## Abstract

**Background/Objectives:** Adequate sleep is critical for adolescents’ physical and mental health. However, academic demands and lifestyle habits lead to insufficient sleep among many adolescents. This study examined the relationship between sleep patterns and general characteristics, health behaviors, and mental health among South Korean adolescents. **Methods:** Data were obtained from the Korea Youth Risk Behavior Survey conducted by the Korea Disease Control and Prevention Agency, involving 21,283 students aged between 13 and 18 years. Self-reported sleep duration was categorized as adequate (8–10 h) or insufficient (less than 8 h on both weekdays and weekends), and 20 independent variables across demographic, socioeconomic, lifestyle, health-related behavioral, and psychological factors were analyzed. Multiple logistic regression analyses were conducted to evaluate the effects of insufficient sleep. **Results:** Insufficient sleep was more prevalent among female students (odds ratio [OR]: 2.064) and older students (OR: 16.588 for Grade 12 vs. Grade 7) and was associated with higher stress levels (OR: 4.338 for almost always vs. never), suicidal ideation (OR: 1.826), and unhealthy behaviors such as alcohol consumption (OR: 2.009), smoking (OR: 1.998), and smartphone overdependence (OR: 2.313 for severe vs. normal). In contrast, adolescents with adequate sleep reported greater happiness (OR: 4.167 for very much vs. not at all) and better academic performance (OR: 1.377 for very high vs. very low). **Conclusions:** The findings show that insufficient sleep significantly affects adolescent well-being, highlighting the need for tailored interventions and increased societal awareness. Future research should explore the mechanisms underlying gender differences and weekday–weekend sleep disparities to enhance sleep quality in this population.

## 1. Introduction

Sleep is a critical determinant of human physical and mental health. However, contemporary lifestyles often compromise sleep duration in favor of managing heavy workloads, pursuing personal development, and making academic preparations. Notably, insufficient sleep disrupts physiological systems, increasing the risk of cardiovascular disease, diabetes, obesity, and cognitive impairment, as well as the incidence of motor vehicle and workplace accidents [1,2].

Adolescence is characterized by rapid physical, psychological, and social development. Thus, adolescents require adequate sleep as a fundamental mechanism for regulating and adapting to these multifaceted transitions [3]. Sufficient sleep during this critical stage is strongly associated with enhanced overall well-being, healthier behavioral patterns, reduced stress levels, and a lower prevalence of obesity [4]. Several studies have shown that deviations from an optimal sleep duration, both shorter and longer, have a more pronounced impact on mental health and quality of life in adolescents than in other age groups [5]. Insufficient sleep often leads to daytime drowsiness, which can substantially impair concentration, academic performance, and psychological stability. Moreover, it affects psychological factors such as fatigue, depression, and irritability [6,7].

Insufficient sleep among adolescents has recently garnered global concern [1,8]. This issue is particularly severe in South Korea, where the dual pressure of rigorous academic demands and pervasive smartphone use, both of which adversely affect sleep quality, result in significant sleep deprivation among adolescents [9]. Compared to their counterparts in other developed countries, South Korean adolescents experience significantly greater sleep deprivation due to academic pressure and extended study hours. According to the OECD’s Health at a Glance report, South Korean adolescents sleep an average of less than 6 h on school nights, whereas their peers in the US, France, and Germany sleep an average of 7–8 h per night [10]. Similarly, while the National Sleep Foundation reported that 57.8% of US adolescents sleep less than 8 h on school nights, this percentage is even higher in South Korea owing to extensive academic demands [11]. Moreover, adolescents with high smartphone dependency exhibit delayed sleep onset, reduced sleep duration, and frequent nighttime awakenings, further exacerbating sleep disturbances [12]. In South Korea, nearly 30% of adolescents are classified as high-risk or at-risk smartphone users, reflecting one of the highest rates of adolescent smartphone overdependence worldwide [13,14].

In South Korea, academic pressure and late-night studying play a predominant role in sleep deprivation, as students often engage in extensive after-school study sessions to prepare for university entrance exams [15]. In contrast, studies from Western countries have indicated that social activities and excessive screen exposure before bedtime are key determinants of insufficient sleep among adolescents [16]. Research has shown that prolonged engagement with digital media, particularly late-night social media use, significantly delays sleep onset and reduces total sleep duration in Western adolescents [12]. However, both South Korean and Western studies have demonstrated that delaying school start times can improve sleep duration and lead to better academic and mental health outcomes, reinforcing the significance of structural interventions across cultural settings. Thus, direct cross-cultural analyses are needed to better understand these sociocultural determinants and provide evidence-based policy recommendations.

Insufficient sleep is strongly associated with heightened emotional instability, elevated levels of depression and stress, and an increased incidence of suicide among adolescents [17,18]. Mental health challenges related to sleep deprivation have become a pressing social issue, with sleep-related factors exerting a profound influence on negative outcomes such as suicide and depression. Alarmingly, suicide in South Korea has recently surpassed traffic accidents as the leading cause of death among adolescents, and the suicide rate has continued to increase steadily [19]. Suicidal ideation, characterized by persistent thoughts about death, serves as a critical predictor of suicidal behavior and an important indicator of adolescents’ mental health status. Although this study identified a significant association between insufficient sleep and adverse mental health outcomes, including increased stress and suicidal ideation, these relationships are likely bidirectional. Elevated stress and psychological distress may also contribute to sleep deprivation, thereby creating a reinforcing cycle.

Adolescence is characterized by rapid physical, mental, and social changes. Insufficient sleep exacerbates these challenges by disrupting one’s ability to regulate and adapt to daily transitions. Consequently, adolescents often attempt to compensate for insufficient sleep on weekends or holidays. However, evidence suggests that compensating for insufficient weekday sleep during the weekend can disrupt sleep patterns, leading to difficulties falling asleep on Sunday nights and perpetuating a cycle of inadequate and irregular sleep [20]. These findings highlight the need to carefully evaluate strategies for mitigating the effects of insufficient sleep.

Adolescents are particularly susceptible to the adverse effects of sleep deprivation, including its associated mental health issues, making them more vulnerable than older age groups [21]. Healthy and sufficient sleep is critical for maintaining mental and physical health in adolescents. However, most studies on sleep duration have focused on adults or older populations, with relatively few taking an integrated approach to examine the multifaceted relationship between sleep duration and adolescent mental health, behavior, and academic performance. Consequently, there is a need to utilize a large-scale, nationally representative dataset to analyze both weekday and weekend sleep patterns in relation to multiple health and behavioral outcomes and to investigate gender differences in the effects of sleep deprivation.

To address these gaps, this study aimed to examine the association between sleep duration and various physical, mental, and behavioral health factors in South Korean adolescents. Specifically, we investigated (1) whether insufficient sleep is associated with increased psychological distress (e.g., stress, anxiety, and suicidal ideation), (2) unhealthy behaviors such as smoking, alcohol consumption, and smartphone overdependence, and (3) whether adequate sleep is positively correlated with better academic performance and overall well-being. Based on previous research, we hypothesized that insufficient sleep is significantly associated with poorer mental health, increased engagement in unhealthy behaviors, and lower academic performance among adolescents.

## 2. Materials and Methods

### 2.1. Study Design

This study utilized data from the 19th Korea Youth Risk Behavior Survey (KYRBS) conducted by the Korea Disease Control and Prevention Agency. The KYRBS is a government-approved statistical survey that has been conducted annually since 2005. The survey aims to assess and monitor health behaviors and trends among Korean adolescents, focusing on topics such as demographics, smoking, alcohol consumption, academic records, smartphone overdependence, mental health, and physical activity. The survey is administered anonymously through a self-reported online questionnaire and targets students in grades 7–12 across South Korea. In 2023, the survey comprised 88 questions covering topics such as smoking, alcohol consumption, and physical activity and produced 92 health indicators. The survey was conducted using a nationally representative sample of 800 schools, including 400 middle schools and 400 high schools. Of these, 799 schools (399 middle schools and 400 high schools) participated, yielding 52,880 respondents, with a response rate of 92.9%. The 52,880 respondents included in the study did not report any chronic diseases, medication use, or sleep disorders that could have influenced their sleep duration and quality.

**Figure 1:**
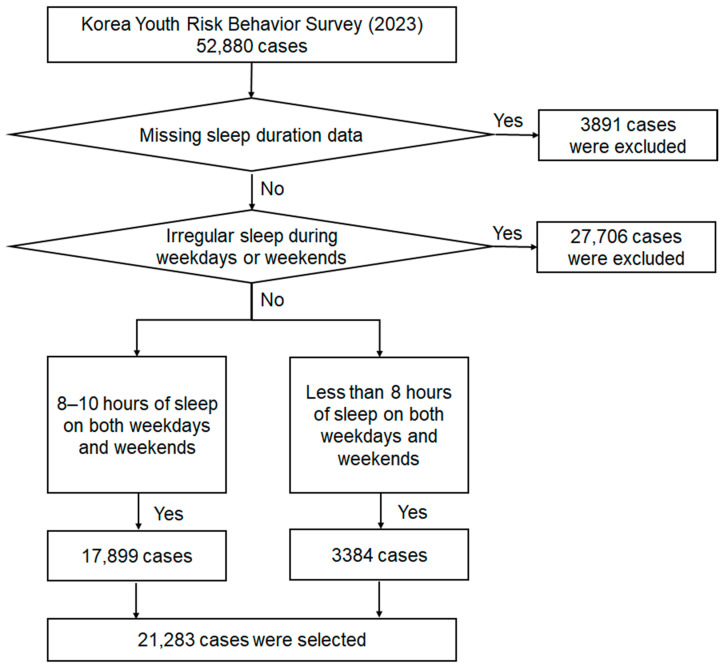
Flowchart depicting the selection of study participants.

For this analysis, weekday and weekend sleep duration data were examined. None of the sleep duration variables had missing values. Adolescents who provided consistent responses for both weekday and weekend sleep duration, categorized as either adequate or insufficient sleep, were included in the final sample for analysis. In total, 21,283 participants met the inclusion criteria. The study protocol was approved by the Institutional Review Board (No: 1041849-202410-BM-214-01) of Yonsei University. Owing to the retrospective nature of this study, the Institutional Review Board waived the need to obtain informed consent. Figure 1 presents a flowchart illustrating the participant selection process.

### 2.2. Study Variables

The dependent variable was sleep duration, defined based on the American Academy of Sleep Medicine (AASM) guidelines for age-appropriate sleep duration in children and adolescents [22]. In the KYRBS, participants reported the time they went to bed and woke up on weekdays (Monday–Friday) and weekends (Saturday–Sunday) separately.

The AASM recommends 8–10 h of sleep per night for adolescents aged 13–18 to support optimal physical and cognitive development [22]. Empirical research has shown that adolescents who sleep less than 8 h per night are at significantly greater risk for psychological distress, cognitive impairment, and engagement in health-risk behaviors [23]. Therefore, participants who reported sleeping 8–10 h per night on both weekdays and weekends were classified as having adequate sleep, whereas those who reported sleeping less than 8 h per night on both weekdays and weekends were classified as having insufficient sleep.

To account for potential confounding factors that could influence the association between sleep duration and adolescent health outcomes, we included a range of demographic, socioeconomic, and behavioral factors in our analysis. These variables were selected based on prior research indicating their potential effects on sleep patterns and mental health in adolescents [24,25].

The independent variables included general, behavioral, and mental health characteristics, based on responses to a self-administered questionnaire. General characteristics included sex, school grade (grades 7–12), district (metropolitan, provinces, others), school type (co-education, boys only, girls only), economic level (quintiles from low to high), residential type (family, relatives, living alone, dormitory, nursery facilities), weekly breakfast frequency, and weekly exercise frequency. Behavioral characteristics included alcohol consumption, smoking status, sexual experience, and suicide-related factors such as suicidal thoughts, plans, and attempts. Academic performance was categorized into five levels, ranging from very low to very high. Smartphone overdependence was assessed using a scale developed by the National Information Society Agency in 2017. This scale consists of 10 items, with total scores ranging from 0 to 40. Participants with scores of 23–30 were classified as the at-risk group for smartphone overdependence, those with scores of 31 or higher as the severe group, and those with scores below 23 as the normal group [26]. The mental health characteristics assessed included subjective happiness, evaluated using a 5-point scale, where higher scores indicated greater happiness; perceived stress, also measured on a 5-point scale, with higher scores indicating greater stress levels; and experiences of sadness, classified as either “none in the past 12 months” or “present in the past 12 months”. Anxiety levels were assessed on a 21-point scale, with higher scores indicating greater anxiety. Anxiety was categorized as normal (0–9 points), at-risk (10–14 points), or severe (15–21 points) [27] based on the participants’ responses to the questionnaire.

### 2.3. Data and Statistical Analysis

We calculated means and standard deviations for continuous outcomes and counts and percentages for categorical outcomes. We used logistic regression models to calculate odds ratios (OR). Logistic regression was chosen because it is well-suited for binary dependent variables, provides an intuitive interpretation via odds ratios, and effectively adjusts for multiple covariates. We reported OR, 95% confidence intervals (95% CI), and beta coefficients. We set the significance level at a 2-sided *p*-value of <0.05. Statistical analyses were performed using IBM SPSS Statistics (version 27; IBM Corp., Armonk, NY, USA) and R version 4.2.0 (R Foundation for Statistical Computing, Vienna, Austria).

## 3. Results

We classified 17,899 students as having insufficient sleep and 3384 as having adequate sleep (Figure 1). As shown in Table 1, the percentage of students aged 13–18 years was higher in the insufficient sleep group (84.1%) than in the adequate sleep group (15.9%). The percentage of male students was lower in the insufficient sleep group (47.0%) and higher in the adequate sleep group (64.7%) compared to the percentages of female students. When comparing students in grade 7 to those in grades 8–12, the percentage of students in the insufficient sleep group increased, whereas that in the adequate sleep group decreased. The percentage of students living in metropolitan areas was higher in the insufficient sleep group (45.5%) than in the adequate sleep group (36.8%). Conversely, the percentages of students living in provinces and other local subdivisions were lower in the insufficient sleep group (47.7% and 6.9%, respectively) than in the adequate sleep group (53.5% and 9.8%, respectively). The percentages of students attending co-educational and boys-only schools were lower in the insufficient sleep group (66.5% and 15.3%, respectively) than in the adequate sleep group (73.9% and 16.6%, respectively). In contrast, the percentage of students attending girls-only schools was higher in the insufficient sleep group (17.7%) than in the adequate sleep group (9.5%). The percentage of students from economically disadvantaged backgrounds was higher in the insufficient sleep group (7.9%) than in the adequate sleep group (6.7%). Conversely, the percentage of students from economically advantaged backgrounds was lower in the insufficient sleep group (13.4%) than in the adequate sleep group (15.6%). The percentage of students who lived with their families was higher in the adequate sleep group (97.6%) than in the insufficient sleep group (94.9%). Students in the adequate sleep group (4.4 ± 2.7 days) had breakfast more frequently than those in the insufficient sleep group (3.6 ± 2.8 days). Daily exercise for a week was more frequently observed in the adequate sleep group (2.6 ± 2.3 days) than in the insufficient sleep group (2.1 ± 2.1 days).

Table 2 presents the number of students with self-reported outcomes, including behaviors (alcohol use, smoking, and sexual experience), suicide-related factors (ideation, planning, and attempts), academic records, and smartphone overdependence for categorical outcomes, along with the means and standard deviations for continuous outcomes. Alcohol consumption was higher in the insufficient sleep group (34.8%) than in the adequate sleep group (21.0%). Similarly, smoking was more common in the insufficient sleep group (9.6%) than in the adequate sleep group (5.1%). More adolescents reported having sexual experience in the insufficient sleep group (7.0%) than in the adequate sleep group (2.9%). More students reported suicidal ideation (15.2%), suicide plans (5.9%), and suicide attempts (3.4%) in the insufficient sleep group than in the adequate sleep group (8.9%, 3.1%, and 2.0%, respectively). Students in the adequate sleep group (15.6%) also tended to have higher academic records than those in the insufficient sleep group (13.4%). Conversely, the lowest academic records were observed more frequently in the insufficient sleep group (7.9%) than in the adequate sleep group (6.7%). Finally, smartphone overdependence was more prevalent in the insufficient sleep group (5.2%) than in the adequate sleep group (2.5%).

As shown in Table 2, the odds of alcohol consumption were 101% higher (OR, 2.009, 95% CI: 1.840–2.195) in the insufficient sleep group than in the adequate sleep group. Similarly, the odds of smoking were 119% higher (OR, 1.998, 95% CI: 1.699–2.348) in the insufficient sleep group compared to the adequate sleep group. The odds of having sexual experience were 149% higher (OR, 2.485, 95% CI: 2.018–3.060) in the insufficient sleep group than in the adequate sleep group. The odds of suicidal ideation were 83% higher (OR, 1.826, 95% CI: 1.611–2.069) in the insufficient sleep group than in the adequate sleep group. The odds of having a suicide plan were 95% higher (OR, 1.954, 95% CI: 1.593–2.396) in the insufficient sleep group than in the adequate sleep group. The odds of attempting suicide were 71% higher (OR, 1.712, 95% CI: 1.329–2.205) in the insufficient sleep group than in the adequate sleep group. The odds of having the highest academic records were 27% lower (OR, 0.726, 95% CI: 0.613–0.860) in the insufficient sleep group than in the adequate sleep group. Finally, the odds of smartphone overdependence were 131% higher (OR, 2.313, 95% CI: 1.845–2.899) in the insufficient sleep group than in the adequate sleep group.

Table 3 presents data related to students’ self-reported mental health status. More students reported experiencing the lowest level of happiness in the insufficient sleep group (2.0%) than in the adequate sleep group (0.8%). Conversely, the highest self-reported happiness was observed in the adequate sleep group (32.0%) compared to the insufficient sleep group (18.5%). Students in the adequate sleep group (4.7%) reported the lowest level of stress more frequently than those in the insufficient sleep group (2.2%). Conversely, the highest self-reported stress was observed in the insufficient sleep group (10.7%) compared to the adequate sleep group (5.3%). Higher levels of sadness were observed in the insufficient sleep group (10.7%) than in the adequate sleep group (5.3%). Finally, the highest self-reported anxiety was found in the insufficient sleep group (5.1%) compared with the adequate sleep group (2.4%).

The odds of the highest self-reported happiness were 76% lower (OR, 0.240, 95% CI: 0.163–0.355) in the insufficient sleep group compared to the adequate sleep group. In addition, the odds of the highest self-reported stress were 334% higher (OR, 4.338, 95% CI: 3.415–5.509) in the insufficient sleep group than in the adequate sleep group. The odds of sadness were 81% higher (OR, 1.811, 95% CI: 1.650–1.989) in the insufficient sleep group compared to the adequate sleep group. Finally, the odds of the highest self-reported anxiety were 131% higher (OR, 2.311, 95% CI: 1.836–2.911) in the insufficient sleep group than in the adequate sleep group.

## 4. Discussion

This study examined the effects of sufficient and insufficient sleep on the lives of South Korean adolescents to emphasize the critical role of sleep during this developmental period and to provide foundational data for promoting adequate sleep. The analysis revealed significant gender differences in sleep duration, with a higher proportion of male students in the adequate sleep group and a lower proportion in the insufficient sleep group, indicating that male students generally achieve better sleep than their female counterparts. This observation aligns with previous studies reporting that female students experience greater resistance to bedtime, shorter sleep durations, and more frequent complaints of insufficient sleep on weekdays [28,29,30,31]. Previous research has suggested that these differences may stem from higher levels of fatigue and depression among female students, as well as greater susceptibility to smartphone overdependence [32,33,34]. These findings highlight the importance of gender-sensitive approaches to sleep education and interventions aimed at addressing disparities in sleep duration.

In addition to psychosocial factors, biological mechanisms may contribute to the negative effects of insufficient sleep. Circadian misalignment, caused by delayed melatonin secretion in adolescents, results in chronic sleep debt and impaired cognitive function and emotional regulation [35]. Adolescents naturally experience a phase delay in melatonin secretion, and early school start times exacerbate this misalignment, leading to excessive daytime sleepiness and cognitive deficits [36]. Moreover, dysregulation of the hypothalamic–pituitary–adrenal (HPA) axis caused by sleep deprivation can lead to increased cortisol levels, thereby exacerbating stress and emotional instability [37]. Chronic activation of the HPA axis is associated with an increased risk of anxiety and depression in adolescents who experience persistent sleep restriction [38]. Furthermore, neurotransmitter imbalances, particularly decreased serotonin and dopamine levels, may explain the increased prevalence of depressive symptoms and risky behaviors observed in adolescents with insufficient sleep [39]. Disruptions in serotonin and dopamine levels caused by sleep deprivation have been linked to impaired impulse control, increased emotional reactivity, and a heightened risk of engaging in unhealthy behaviors, such as substance use and smartphone overdependence [40]. These biological pathways underscore the multifaceted impact of insufficient sleep and highlight the need for interventions that address both the behavioral and physiological determinants of adolescent sleep health.

The analysis also revealed a distinct trend of decreasing sleep adequacy as students advanced in grades. The proportion of students who received sufficient sleep decreased significantly in high school, accompanied by a marked decline in sleep duration. This trend can be attributed to increasing academic demands and early school start times, which conflict with adolescents’ biological rhythms. Studies have indicated that melatonin secretion, a key regulator of sleep, is delayed in adolescents, typically beginning at approximately 11:00 PM. Consequently, early school start times result in residual melatonin levels upon waking, impairing alertness and cognitive performance [30,35,36,41]. Chronic insufficient sleep among adolescents is associated with a reduced ability to retain new information and poorer academic outcomes.

Research conducted in other contexts supports the effectiveness of delaying school start times to mitigate these issues. For example, the American Academy of Pediatrics recommended a 30 min delay in school start times to significantly increase the proportion of students who can receive sufficient sleep [42]. Furthermore, this adjustment can positively influence mental health, academic performance, and overall quality of life [42,43]. These findings underscore the urgent need for national-level interventions to address insufficient sleep among adolescents by reconsidering early school start times and implementing policies that better align with adolescents’ sleep needs.

Given the widespread consequences of insufficient sleep among adolescents, policy-level interventions are critical for mitigating its adverse effects. Delaying school start times is a well-supported intervention, as research has consistently shown that even modest delays (e.g., 30–60 min) can increase sleep duration, improve academic performance, and enhance mental health outcomes for adolescents [36]. For example, the American Academy of Pediatrics strongly recommends that middle and high schools start no earlier than 8:30 AM to better align with adolescent circadian rhythms [44]. Despite these recommendations, their implementation remains inconsistent, highlighting the need for policy advocacy at the national level. Furthermore, integrating comprehensive sleep education programs into school curricula may help adolescents and their families develop healthier sleep habits. Prior studies have indicated that structured sleep education interventions, including behavioral strategies for improving sleep hygiene, can lead to long-term benefits in adolescent sleep quality and mental well-being [45]. Moreover, public health campaigns targeting the risks of excessive screen time before sleep and promoting consistent sleep schedules could further contribute to improving adolescent sleep behaviors. By incorporating such evidence-based policies, governments and educational institutions can play crucial roles in addressing the growing issue of sleep deprivation among adolescents.

In metropolitan areas, more adolescents experienced insufficient sleep than adequate sleep. Conversely, in small cities and rural areas, adolescents with adequate sleep were more prevalent. Despite these findings, previous studies have reported mixed results regarding the relationship between sleep quality and urban versus rural settings. For example, poorer sleep quality was observed in urban areas in Mexico, whereas similar changes were found in rural areas in China [46,47].

Adolescents attending co-educational or boys-only schools were more likely to receive adequate sleep than those attending girls-only schools. This observation aligns with existing research indicating that male students generally achieve better sleep than female students. However, other studies have highlighted the significant influence of pre-sleep behaviors on insufficient sleep, regardless of school type [48].

Sleep duration can also be influenced by socioeconomic status and living conditions. In this study, adolescents from middle-upper and upper economic levels, as well as those living with their families, were more likely to achieve adequate sleep. These results are consistent with those of a US-based study that reported that adolescents from lower-income households experienced poorer subjective sleep quality and greater daytime drowsiness [49].

The two sleep groups were further differentiated by lifestyle. Adolescents with adequate sleep were more likely to have breakfast daily and engage in regular physical activity, whereas insufficient sleep was associated with behaviors such as skipping breakfast and reduced exercise [50]. Additionally, studies on Japanese adolescents have revealed that skipping breakfast, poor sleep quality, and unhealthy lifestyle choices are linked to lower subjective happiness [51]. In contrast, studies from Italy and the US reported no significant relationship between sleep habits and general behavioral characteristics, emphasizing the role of sociocultural differences [52].

Academic achievement was significantly higher among adolescents with adequate sleep. Insufficient sleep has been associated with declines of 11–18% in mathematics and science performance, whereas severe sleep disturbances have been linked to heightened anxiety, which can occasionally coincide with improved performance under high-pressure conditions [53,54]. Furthermore, studies on college students have highlighted a U-shaped relationship between sleep duration and academic outcomes, suggesting that both insufficient and excessive sleep may be associated with differences in learning outcomes [6].

Health behaviors, such as alcohol consumption, smoking, and smartphone overdependence, were more prevalent in the insufficient sleep group. High-risk smartphone use has been found to be particularly associated with lower sleep satisfaction among South Korean adolescents [55]. Similarly, research on young adults has linked nicotine dependence, substance use, and smartphone addiction to poorer sleep quality [56,57,58]. These findings underscore the importance of targeted interventions by families and schools to regulate smartphone use and foster healthy sleep habits among adolescents.

Insufficient sleep among South Korean adolescents has been partially attributed to smartphone overdependence, which plays a significant role in their daily lives and well-being. Although smartphones are indispensable tools for communication and entertainment, their excessive use often leads to sleep deprivation, which can lead to severe long-term adverse effects. Addressing this issue requires proactive guidance from families and schools to help adolescents regulate their smartphone use. A longitudinal study by Taylor et al., which tracked adolescents aged 12–18 years over 6–7 years, revealed that insufficient sleep during adolescence is associated with higher rates of alcohol and marijuana use, illegal drug consumption, suicidal ideation, and suicide attempts, as well as an increased risk of depression in adulthood [59]. Given the profound influence of adolescent health behaviors and conditions on later health and quality of life, sufficient sleep should be the cornerstone of health promotion and disease prevention strategies.

Adolescents in the adequate sleep group reported significantly higher levels of happiness than their peers in the insufficient sleep group. In contrast, stress, anxiety, and suicidal ideation, planning, and attempts were markedly higher among adolescents with insufficient sleep. These findings are consistent with those of previous research linking insufficient sleep to reduced subjective happiness, heightened stress, and an increased prevalence of depression among middle and high school students [60,61]. These findings underscore the direct relationship between insufficient sleep and emotional challenges, including anxiety, stress, and suicidal tendencies. Although this study identified an association between sleep deprivation and mental health outcomes, the psychological and physiological mechanisms underlying this relationship remain unclear. Previous research has suggested that inadequate sleep may influence emotional regulation, stress responses, and neurobiological processes linked to mental well-being [62,63]. However, further longitudinal and experimental studies are needed to identify the causal pathways and neurophysiological underpinnings of this relationship. Furthermore, prior studies have identified a range of factors that influence adolescents’ mental health, such as gender, academic achievement, socioeconomic status, anxiety, depression, sleep disorders (e.g., insomnia), alcohol consumption, drug use, smartphone overdependence, and suicide-related behaviors [31,64].

Adolescence is a particularly vulnerable period for emotional stability because of the psychological stress associated with rapid physical and developmental changes [60,65]. Ensuring adequate sleep duration and improving sleep quality are critical for supporting mental health during this formative stage. Promoting healthy sleep habits tailored to adolescents’ developmental needs through targeted policies and comprehensive social initiatives is essential for achieving these goals.

This study has identified areas for further improvement and exploration. Although gender differences in sleep patterns were observed, the specific mechanisms underlying these differences were not thoroughly examined. Future studies should integrate objective sleep measures, such as actigraphy or polysomnography, to enhance the accuracy of gender-based sleep analyses. As evidence suggests that female adolescents are more prone to sleep disturbances and sleep-related emotional distress than their male counterparts [66], longitudinal research exploring the hormonal, behavioral, and psychosocial contributors to these differences is essential. In addition, reliance on self-reported data may have introduced recall bias or inaccuracies, potentially affecting the validity of the findings. Future studies should address this issue by integrating multi-modal assessment approaches to enhance measurement reliability. Furthermore, this study categorized participants based on adequate or insufficient sleep across both weekdays and weekends without distinguishing the unique effects of weekday versus weekend sleep durations. Future research should systematically assess weekday and weekend sleep patterns to better evaluate sleep quality and its effects on adolescents. The efficacy of sleep compensation strategies, such as weekend catch-up sleep and daytime napping, remains unclear and warrants further investigation. Controlled sleep extension studies could determine whether these behaviors restore cognitive and emotional function [67]. Additionally, accounting for individual differences in chronotypes and sleep regulation will refine intervention strategies. These methodological advances will enhance our understanding of the complex interplay between sleep deprivation, gender differences, and adaptive coping mechanisms. Finally, although this study primarily examined the relationship between sleep duration and adolescent well-being, sleep quality is a critical factor that may influence mental health, behavior, and academic performance. Future research should incorporate objective sleep quality measures (e.g., sleep latency, nighttime awakenings, and sleep efficiency) to provide a more comprehensive understanding of adolescent sleep health.

This study also had limitations regarding the measurement of exercise-related variables. Owing to the structure of the KYRBS dataset, we could only assess exercise frequency on weekdays, without accounting for exercise duration or intensity. As both factors are key determinants of physical health and well-being, future research should incorporate more detailed exercise parameters to develop a better understanding of the relationship between physical activity, sleep, and adolescent health outcomes. This study relied on self-reported sleep duration and health behaviors, which may have introduced recall and social desirability biases. Adolescents may misestimate their sleep duration or underreport unhealthy behaviors, such as smoking and smartphone overuse. Future research should incorporate objective sleep measures (e.g., actigraphy and polysomnography) and behavioral tracking to improve data accuracy. Despite these limitations, self-reported sleep duration remains a widely used and valuable tool in large-scale epidemiological studies. Additionally, although this study accounted for several demographic and behavioral variables, potential unmeasured confounding factors may have influenced the observed associations. Variables such as socioeconomic background, underlying health conditions, and environmental influences may contribute to differences in sleep patterns and mental health outcomes. Future research should employ more comprehensive statistical adjustments and longitudinal or experimental designs to improve causal inferences and provide more robust results. Furthermore, although this study provided valuable insights into the association between sleep duration and adolescent health outcomes, it did not explicitly incorporate a theoretical framework. Our primary objective was to conduct an empirical analysis using a large-scale, nationally representative dataset. However, future research could benefit from applying models such as the biopsychosocial model or self-regulation theory to better contextualize the mechanisms linking insufficient sleep to mental health, behavioral outcomes, and academic performance. Integrating such frameworks may provide a more nuanced understanding of these associations and inform targeted interventions for improving adolescent sleep health.

## 5. Conclusions

This study investigated the relationship between sleep patterns and the general characteristics, health behaviors, and subjective mental health of South Korean adolescents. Although this study identified strong associations between insufficient sleep and adverse health outcomes through logistic regression analysis, its cross-sectional nature limits causal inferences. Nevertheless, the findings indicate that, as students advance in school, the likelihood of receiving adequate sleep declines significantly. Insufficient sleep was found to be associated with diminished emotional stability, increased stress, heightened depression, suicidal ideation, and a higher prevalence of behaviors detrimental to health. These results emphasize the essential role of adequate sleep in supporting adolescents’ overall well-being, including their physical and mental health and behavioral patterns. These findings highlight the need for tailored education and guidance for adolescents to encourage healthy sleep habits. Raising public awareness of the growing issue of declining sleep quality among adolescents is critical, as is addressing gender differences in sleep patterns to develop more effective interventions.

Addressing adolescent sleep deprivation requires a multifaceted approach involving parents, schools, and policymakers. Parents should encourage consistent sleep schedules, limit screen exposure before bedtime, and create a conducive sleep environment at home. Schools can support adolescent sleep health by integrating sleep education programs into their curricula and reconsidering early school start times to align with adolescents’ biological sleep needs. At the policy level, national guidelines promoting later school start times and public health campaigns emphasizing the risks of insufficient sleep could drive systemic changes. Implementing these evidence-based interventions could help mitigate the adverse effects of sleep deprivation and improve adolescents’ cognitive, emotional, and overall well-being.

## Figures and Tables

**Table 1:** Demographics and general information for groups with insufficient and adequate sleep among adolescent students.

	No./Total No. (%), by Group				
	Insufficient Sleep (*n* = 17,899)	Adequate Sleep (*n* = 3384)	*p*-Value	OR (95% CI)	Beta
Sex, Female, *N* (%)	9483 (53.0)	1195 (35.3)	<0.0001 *	2.064 (1.912–2.228)	0.725
School grade, *N* (%)					
Grade 7	2080 (11.6)	1472 (43.5)	Reference	Reference	Reference
Grade 8	2536 (14.2)	889 (26.3)	<0.0001 *	2.019 (1.824–2.234)	0.703
Grade 9	2908 (16.2)	551 (16.3)	<0.0001 *	3.735 (3.336–4.181)	1.318
Grade 10	3599 (20.1)	187 (5.5)	<0.0001 *	13.620 (11.590–16.007)	2.612
Grade 11	3471 (19.4)	144 (4.3)	<0.0001 *	17.058 (14.255–20.413)	2.837
Grade 12	3305 (18.5)	141 (4.2)	<0.0001 *	16.588 (13.838–19.885)	2.809
District, *N* (%)					
Metropolitan	8137 (45.5)	1244 (36.8)	Reference	Reference	Reference
Provinces	8535 (47.7)	1809 (53.5)	<0.0001 *	0.721 (0.667–0.780)	−0.327
Others	1227 (6.9)	331 (9.8)	<0.0001 *	0.567 (0.495–0.649)	−0.568
School type, *N* (%)					
Co-education	11908 (66.5)	2500 (73.9)	Reference	Reference	Reference
Boys only	2815 (15.7)	562 (16.6)	0.326	1.052 (0.951–1.163)	0.050
Girls only	3176 (17.7)	322 (9.5)	<0.0001 *	2.071 (1.832–2.341)	0.728
Economy, *N* (%)					
Low	1420 (7.9)	227 (6.7)	Reference	Reference	Reference
Low-middle	3981 (22.2)	679 (20.1)	0.433	0.937 (0.797–1.102)	−0.065
Middle	5336 (29.8)	976 (28.8)	0.090	0.874 (0.748–1.021)	−0.135
Middle-high	4768 (26.6)	975 (28.8)	0.002 *	0.782 (0.669–0.914)	−0.246
High	2394 (13.4)	527 (15.6)	<0.0001 *	0.726 (0.613–0.860)	−0.320
Resident type, *N* (%)					
Family	16,979 (94.9)	3303 (97.6)	Reference	Reference	Reference
Relatives	88 (0.5)	6 (0.2)	0.013 *	2.853 (1.247–6.529)	1.048
Living alone	103 (0.6)	13 (0.4)	0.142	1.541 (0.865–2.748)	0.433
Dormitory	684 (3.8)	42 (1.2)	<0.0001 *	3.168 (2.315–4.336)	1.153
Nursery facilities	45 (0.3)	20 (0.6)	0.002 *	0.438 (0.258–0.742)	−0.826
Breakfast, days	3.6 ± 2.8	4.4 ± 2.7	<0.0001 *	0.877 (0.875–0.900)	−0.119
Day exercise, days	2.1 ± 2.1	2.6 ± 2.3	<0.0001 *	0.898 (0.883–0.912)	−0.108

Values are presented as means ± standard deviations (SD) for continuous variables and as counts (n) and percentages (%) for categorical variables. Odds ratios (OR) with 95% confidence intervals (CI) and corresponding beta coefficients from the logistic regression models are provided. An asterisk represents a significant difference between groups. OR = odds ratio; CI = confidence interval.

**Table 2:** Comparisons of self-reported outcomes.

	No./Total No. (%), by Group				
	Insufficient Sleep (*n* = 17,899)	Adequate Sleep (*n* = 3384)	*p*-Value	OR (95% CI)	Beta
Behavior, *N* (%)					
Alcohol, *N* (%)	6220 (34.8)	709 (21.0)	<0.0001 *	2.009 (1.840–2.195)	0.698
Smoking, *N* (%)	1720 (9.6)	171 (5.1)	<0.0001 *	1.998 (1.699–2.348)	0.692
Sexual experience, *N* (%)	1247 (7.0)	99 (2.9)	<0.0001 *	2.485 (2.018–3.060)	0.910
Suicide, *N* (%)					
Ideation, *N* (%)	2716 (15.2)	32 (8.9)	<0.0001 *	1.826 (1.611–2.069)	0.602
Plan, *N* (%)	1054 (5.9)	105 (3.1)	<0.0001 *	1.954 (1.593–2.396)	0.670
Attempt, *N* (%)	607 (3.4)	68 (2.0)	<0.0001 *	1.712 (1.329–2.205)	0.538
Academic records, *N* (%)					
Very low	1420 (7.9)	227 (6.7)	Reference	Reference	Reference
Low	3981 (22.2)	679 (20.1)	0.433	0.937 (0.797–1.102)	−0.065
Moderate	5336 (29.8)	976 (28.8)	0.090	0.874 (0.748–1.021)	−0.135
High	4768 (26.6)	975 (28.8)	0.002 *	0.782 (0.669–0.914)	−0.246
Very high	2394 (13.4)	527 (15.6)	<0.0001 *	0.726 (0.613–0.860)	−0.320
Smartphone overdependence, N (%)					
Normal	12,698 (70.9)	2684 (79.3)	Reference	Reference	Reference
At risk	4271 (23.9)	615 (18.2)	<0.0001 *	1.468 (1.336–1.613)	0.384
Severe	930 (5.2)	85 (2.5)	<0.0001 *	2.313 (1.845–2.899)	0.838

Values are presented as means ± standard deviations (SD) for continuous variables and as counts (n) and percentages (%) for categorical variables. Odds ratios (OR) with 95% confidence intervals (CI) and corresponding beta coefficients from the logistic regression models are provided. An asterisk represents a significant difference between groups. OR = odds ratio; CI = confidence interval.

**Table 3:** Comparisons of self-reported mental health outcomes between groups.

	No./Total No. (%), by Group				
	Insufficient Sleep (*n* = 17,899)	Adequate Sleep (*n* = 3384)	*p*-Value	OR (95% CI)	Beta
Self-reported mental health					
Happiness					
Not at all	356 (2.0)	28 (0.8)	Reference	Reference	Reference
Not much	2077 (11.6)	233 (6.9)	0.088	0.701 (0.466–1.054)	−0.355
Neutral	5667 (31.7)	765 (22.6)	0.007 *	0.583 (0.394–0.862)	−0.540
Somewhat	6485 (36.2)	1274 (37.6)	<0.0001 *	0.400 (0.271–0.591)	−0.915
Very much	3314 (18.5)	1084 (32.0)	<0.0001 *	0.240 (0.163–0.355)	−1.425
Stress					
Never	392 (2.2)	160 (4.7)	Reference	Reference	Reference
Rarely	2165 (12.1)	724 (21.4)	0.053	1.221 (0.997–1.494)	0.199
Every once in a while	7896 (44.1)	1611 (47.6)	<0.0001 *	2.001 (1.652–2.423)	0.693
Sometimes	5533 (30.9)	709 (21.0)	<0.0001 *	3.185 (2.608–3.890)	1.159
Almost always	1913 (10.7)	180 (5.3)	<0.0001 *	4.338 (3.415–5.509)	1.467
Sadness, *N* (%)	5084 (28.4)	608 (18.0)	<0.0001 *	1.811 (1.650–1.989)	0.594
Anxiety, scores					
Normal	15,270 (85.3)	3152 (93.1)	Reference	Reference	Reference
At risk	1722 (9.6)	151 (4.5)	<0.0001 *	2.354 (1.985–2.792)	0.856
Severe	907 (5.1)	81 (2.4)	<0.0001 *	2.311 (1.836–2.911)	0.838

Values are presented as means ± standard deviations (SD) for continuous variables and as counts (n) and percentages (%) for categorical variables. Odds ratios (OR) with 95% confidence intervals (CI) and corresponding beta coefficients from the logistic regression models are provided. An asterisk represents a significant difference between groups. OR = odds ratio; CI = confidence interval.

## Data Availability

The data presented in this study are available upon request from the corresponding author. The data are not publicly available because they consist of raw data that have been processed and analyzed for the purposes of this study.

## Abbreviations

The following abbreviations are used in this manuscript:

SD	Standard deviation
OR	Odds ratio
CI	Confidence interval
